# Indexed PCR Primers Induce Template-Specific Bias in Large-Scale DNA Sequencing Studies

**DOI:** 10.1371/journal.pone.0148698

**Published:** 2016-03-07

**Authors:** James L. O’Donnell, Ryan P. Kelly, Natalie C. Lowell, Jesse A. Port

**Affiliations:** 1 School of Marine and Environmental Affairs, University of Washington, Seattle, Washington, United States of America; 2 Center for Ocean Solutions, Woods Institute for the Environment, Stanford University, Stanford, California, United States of America; Central Michigan University, UNITED STATES

## Abstract

Massively parallel sequencing is rapidly emerging as an efficient way to quantify biodiversity at all levels, from genetic variation and expression to ecological community assemblage. However, the number of reads produced per sequencing run far exceeds the number required per sample for many applications, compelling researchers to sequence multiple samples per run in order to maximize efficiency. For studies that include a PCR step, this can be accomplished using primers that include an index sequence allowing sample origin to be determined after sequencing. The use of indexed primers assumes they behave no differently than standard primers; however, we found that indexed primers cause substantial template sequence-specific bias, resulting in radically different profiles of the same environmental sample. Likely the outcome of differential amplification efficiency due to primer-template mismatch, two indexed primer sets spuriously change the inferred sequence abundance from the same DNA extraction by up to 77.1%. We demonstrate that a double PCR approach alleviates these effects in applications where indexed primers are necessary.

## Introduction

The plummeting cost of DNA sequencing has led to the widespread adoption of DNA sequence-based approaches to a wide variety of biological problems [1–4]. An increasingly popular technique for identifying the biological variants (organisms or alleles) present in a sample comprised of template DNA from multiple sources (taxa, genomes, or gene copies) is the parallel sequencing of nucleotide fragments generated by PCR (amplicons) [5, 6]; this approach has seen application to problems such as the bulk identification of organisms either in a combined tissue (e.g. [7–9]) or environmental samples (e.g. [10, 11]). While the per-nucleotide cost of sequencing has dropped, the cost per run remains substantial, and a single run provides many more sequences than is typically required by such amplicon-based studies [6]. Investigators can maximize cost efficiency by sequencing more than one sample on a single run (multiplex sequencing), but only if sequences can be traced back to their sample of origin.

A common solution to this problem is to add unique synthetic oligonucleotide index sequences to the end of DNA strands in each sample, by which samples can be distinguished after sequencing [12]. Conflicting terminology exists for such sequences: they are interchangeably referred to as “barcodes”, “tags”, and “indexes”. “Barcode” is a poor choice because it is already used by biologists to refer to a region of the genome that distinguishes among taxa. “Tag” is slightly better, though may cause confusion with expressed sequence tags. Further, its general definition lacks reference to the linking of disjunct information—sequence and sample—and may instill a false sense of passivity about the sequence. “Index” most adequately captures the purpose of these sequences: to link the sequences produced by parallel sequencing back to the sample from which they originate.

Index sequences can be added to each sample via ligation after PCR during sequencing library preparation; however, these library indexes can be costly and often require proprietary kits. The comparatively low cost of synthesizing oligonucleotides has attracted investigators to a primer-based approach to sample indexing. Index sequences can be included on the 5′ end of the forward and reverse PCR primers, and each sample amplified with a unique set of these indexed primers [3, 13–15]. While the 3′ end of the primer influences primer binding and PCR efficiency far more than the 5′ end [16], there is a danger that using slightly different primers in each sample could yield differences in amplification among samples and among templates in mixed-template samples.

The use of indexed primers assumes that the index portion of the primer does not interact with the template DNA targeted for amplification; however, this assumption may be violated, especially in PCR of template DNA from mixed sources. Mismatches between primer and template reduce the amplification efficiency of PCR [16–20], and in PCRs of mixed templates, this results in over-representation of template sequences without mismatches [17, 19, 21, 22]. An indexed primer is simply a longer oligonucleotide primer, and thus templates with mismatches should yield a lower proportion of amplicons in the final product than those without mismatches. This mechanism would introduce substantial bias especially in cases where target templates derive from diverse organisms, such as samples used for metabarcoding and metagenomics.

Berry and colleagues [23] reported bias introduced by primer indexes and provide a solution by way of a double PCR procedure in which the amplicons from a non-indexed PCR are used as template in a second, indexed PCR. Their study emphasized the effect on variable terminal restriction fragment length polymorphism (T-RFLP), but they also showed that indexed primers influence the reproducibility and results of community-level metrics from 454 pyrosequencing. However, the effect size was small, perhaps because their study focused on a relatively restricted taxonomic group (bacterial communities in the mouse gut) which may have low diversity in the primer-binding region. Numerous subsequent publications have cited the original indexed primer description [13]; but without using the double PCR approach [23], the data presented by these studies are highly likely to suffer from bias. Likewise, several methodological reviews cite the utility of indexed primers without acknowledging the importance of double PCR for mixed template samples. Here, we specifically address the effect of indexed primers on both taxon-specific and community-wide metrics of a single environmental sample. We emphasize that our results are derived from replicate PCRs drawn from a single environmental sample, and that the effect is repeated across environmental samples.

## Methods

### Environmental Sampling

Eleven seawater samples were collected serially over four days from the same location in an eelgrass (*Zostera marina*) bed in Puget Sound, Washington, USA just below the water surface in 1L Nalgene bottles. No specific permission was required to take these samples, consistent with the public nature of marine waters under U.S. and relevant state laws; the studies also involved neither endangered nor otherwise-protected species. Sampling equipment was sterilized before use with a 5-minute soak in 10% bleach solution, followed by thorough rinsing with deinonized water. Each sample was vacuum-filtered within hours onto a cellulose acetate membrane (47 mm diameter; 0.45 *μ*m pore size) in the laboratory, and membranes were stored in Longmire solution at room temperature until DNA extraction [24]. As a negative control for the filtration process, we used the same filtration protocol on deionized water. DNA was extracted using the phenol-chloroform protocol described by Renshaw and colleagues [24].

### Molecular Laboratory Methods

We designed a novel set of primers using ecoPrimers [25] to amplify approximately 115bp of mitochondrial 16S DNA from metazoans exclusively. These primers effectively amplify DNA in a broad array of metazoans, including representatives from Vertebrata, Arthropoda, Mollusca, Echinodermata, Nemertea, and others; their sequences are as follows: 16s_Metazoa_fwd AGTTACYYTAGGGATAACAGCG; 16s_Metazoa_rev CCGGTCTGAACTCAGATCAYGT.

We used the program OligoTag [26] to generate 25 unique DNA sequences to serve as primer indexes (S1 Table). These sequences consisted of 6 nucleotides each, and differed by a minimum Hamming distance of at least three. These were appended to the 5′ end of both the forward and reverse primer sequences, and preceded by 3 ambiguous nucleotides (NNN). The ambiguous nucleotides not only guard against degradation of the index sequence itself, they increase diversity during initial sequencing cycles, which improves identification of clusters on the sequencing substrate (flow cell) and thus enhances the number of reads per run [27]. Thus, each indexed primer consisted of 3bp ambiguities, a unique 6bp index sequence, and a core primer sequence (S1 Table). The same index sequence was appended to both the forward and reverse primer sets to avoid problems associated with dual-indexed multiplexing [28]. Primers were obtained from Integrated DNA Technologies (Coralville, IA, USA).

**Fig 1:**
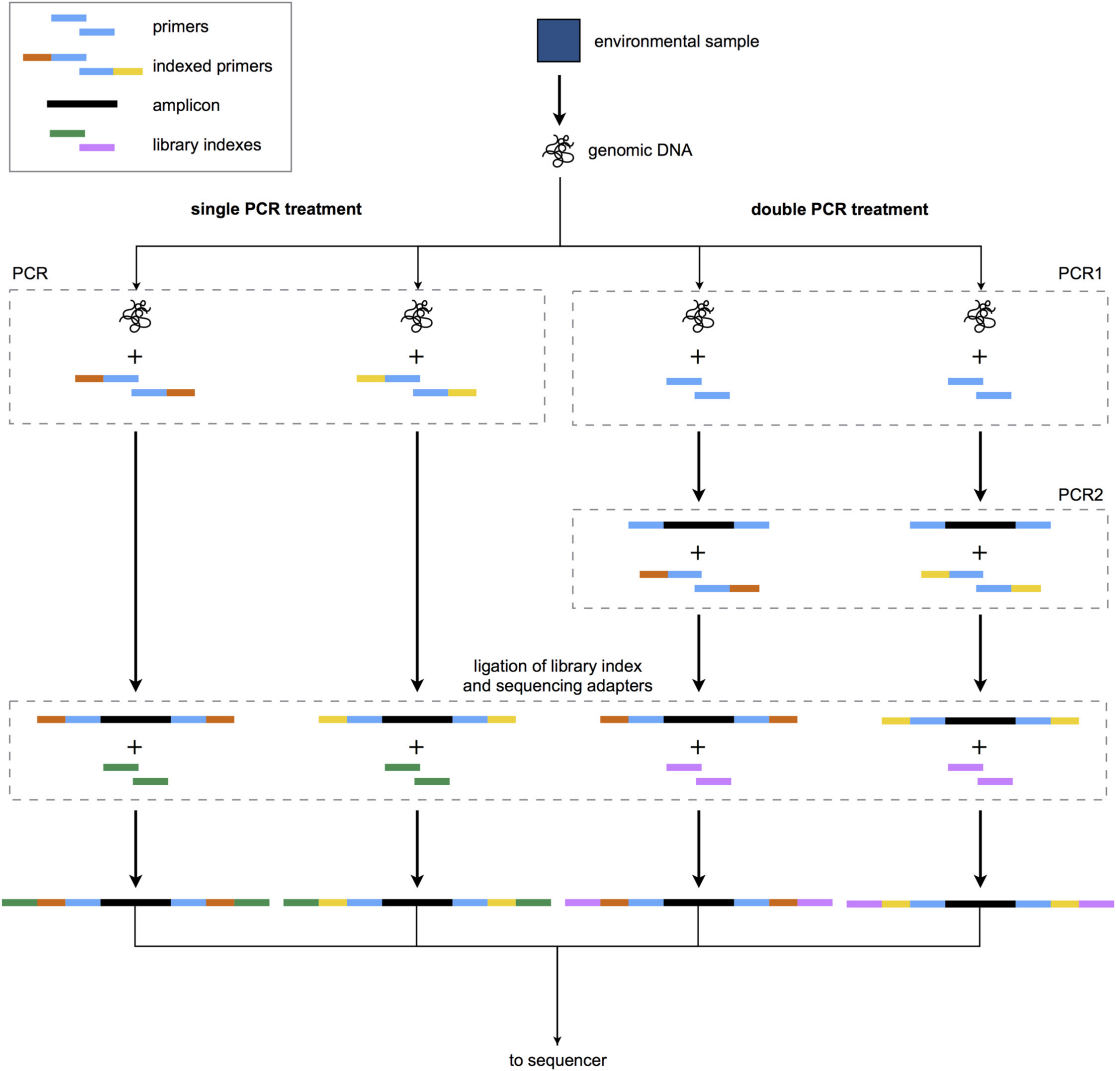
Schematic illustration of study design. Differently colored primer and library indexes represent unique index sequences used to identify the sample origin of reads generated after sequencing on an Illumina MiSeq. A total of 25 indexed primer sets were used, and the single and double PCR treatments were sequenced in separate sequencing runs comprised of three and two uniquely indexed libraries, respectively.

We generated PCR amplicons for sequencing using either a single PCR or a double PCR procedure, illustrated in Fig 1. The single PCR treatment consisted of only a single PCR using indexed primers. The double PCR treatment consisted of a first PCR using non-indexed primers and genomic template (“PCR1”); the diluted products of this reaction were used as template for a second PCR using indexed primers (“PCR2”). All PCRs consisted of the following: 0.25 *μ*L Qiagen HotStar Taq Polymerase, 2.5 *μ*L Qiagen 10x buffer, 0.625 *μ*L (8mM) deoxynucleotide solution, 1 *μ*L (10 *μ*M) each primer (forward and reverse), 18.375 *μ*L water, and 1.25 *μ*L template at 1:100 dilution. PCR volumes larger than 25 *μ*L produced erratic results; therefore, we performed as many 25 *μ*L reactions as needed to generate enough PCR product for each sample, and pooled identical reactions following PCR. A PCR using PCR-grade water in place of template DNA was run along with each batch of PCRs to serve as a negative control against spurious amplification. As a positive control for the PCR and sequencing protocols, we used DNA extracted from a tissue sample of a species absent from the sampled environment (*Oreochromis niloticus*).

The protocols for the single PCR and the first step of the double PCR were essentially identical except for the primers used: non-indexed primers were used in the first step of the double PCR protocol. PCR thermal profiles began with an initialization step (95°C; 15 min) followed by 40 cycles of denaturation (95°C; 15 sec), annealing (61°C; 30 sec), and extension (72°C; 30 sec). Following the single-step PCR, all products generated from the same indexed primer set were combined and purified using the Qiagen MinElute PCR Purification Kit (Qiagen, Hilden, Germany).

For the double PCR protocol we aimed to remove non-indexed primers and spuriously amplified fragments between PCR1 and PCR2. Thus, we pooled five replicate PCR1 products and isolated the target fragment using the AxyPrep Mag FragmentSelect-I kit with solid-phase reversible immobilization (SPRI) paramagnetic beads at 2.5x the volume of PCR product (Axygen BioSciences, Corning, NY, USA). A 1:5 dilution of the resulting solution was used as template for PCR2. PCR2 was otherwise identical to PCR1 except that indexed primers were used and the number of cycles was reduced to 20. Following PCR2, the same SPRI fragment selection protocol was repeated on the pooled products of PCR1.

For the PCR controls and a random sample of field samples, we visualized 5 *μ*L PCR product on a 2% agarose to confirm amplicons were absent from the negative controls, and of the correct size from the field samples. No negative controls produced amplicons. The concentration of double-stranded DNA was quantified from a 2 *μ*L sample using a QuBit fluorometer with the dsDNA HS assay (Life Technologies, Carlsbad, CA, USA).

For the single PCR experiment, the 25 samples (11 environmental samples × 2 indexed primer sets, 2 positive controls × 2 indexed primer sets, 1 negative control) were pooled at equal concentration and then divided into three equal aliquots for individual library preparation, resulting in 75 samples total.

For the double PCR treatment, each of the 11 environmental samples were amplified in a total of four reactions, twice with each of two distinct indexed primer sets, while controls were amplified in three reactions with distinct indexed primer sets. The replicates from each DNA sample were kept separate throughout library preparation. We created two pools of 25 samples (11 environmental samples × 2 indexed primer sets, 1 positive or negative control × 3 indexed primer sets) at equal concentrations for individual library preparation, resulting in 50 samples total.

Pooled samples (150 ng) were prepared for library sequencing using the KAPA high-throughput library prep kit with real-time library amplification protocol (KAPA Biosystems, Wilmington, MA, USA). An index sequence to distinguish libraries along with proprietary adapter sequences that bind the amplicons to the sequencer flow cell were ligated onto the libraries using NEXTflex DNA barcodes (BIOO Scientific, Austin, TX, USA). Libraries were 150bp paired-end sequenced on an Illumina MiSeq at the Stanford Functional Genomics Facility, where 20% PhiX Control v3 was added to act as a sequencing control and to enhance sequencing depth.

### Sequence Processing

Forward and reverse reads were merged using PEAR v0.9.4 [29] and discarded if more than 0.01 of the bases were uncalled. If a read contained two consecutive base calls with quality scores less than 15 (i.e. probability of incorrect base call = 0.0316), these bases and all subsequent bases were removed from the read. Paired reads for which the probability of matching by chance alone exceeded 0.01 were not assembled and omitted from the analysis. Assembled reads were discarded if assembled sequences were not between 50 and 168 bp long, or if reads did not overlap by at least 100 bp.

Merged reads were discarded if the sum of the per-base error probabilities was greater than 0.5 (“expected errors” USEARCH v7.0.1090 [30]. Sequences were demultiplexed on the basis of the 6bp index sequence at base positions 4–9 at both ends using the programming language AWK. Primer sequences were removed using cutadapt v1.7.1 [31], allowing for 2 mismatches in the primer sequence. To speed up subsequent clustering, identical sequences were consolidated in python. Singleton sequences were removed. Sequences were clustered into operational taxonomic units (OTUs) using usearch v7.0.1090 with a clustering radius of 1%, and chimeric sequences were removed [30]. The final data are thus a matrix of counts of OTUs present in each sample.

### Data Analysis

We assessed bias by calculating the mean pairwise Bray-Curtis dissimilarity of OTU sequence counts derived from the same environmental samples (*N* = 11) at two levels of replication: primer index replicates and library replicates. If the counts of sequences for all OTUs in two replicates are identical, their Bray-Curtis dissimilarity is 0; if they are completely dissimilar, their Bray-Curtis dissimilarity is 1. If primer indexes cause variable amplification efficiency among different template DNA in the PCR, the resulting OTU sequence counts will differ among primer index reactions performed on the same environmental sample. Thus, we expect that primer index and library replicates of the same environmental sample should have mean dissimilarity close to 0. Further, if there is no effect of primer index on the OTU sequence counts, there should be no difference between the mean Bray-Curtis dissimilarities calculated among primer index replicates and among library replicates. Analyses were conducted in the statistical programming environment R v3.1.1 [32] and the package vegan [33].

## Results

Both sequencing runs resulted in a large number of high-quality sequences (single PCR = 13,200,683; double PCR = 16,635,743), with similar distributions of reads per sample, although with expected differences due to the number of samples per run (single PCR = 149,071.2±57,944.15, *N* = 75; double PCR = 268,923.2±164,941.9, *N* = 50). A total of 24100 and 35909 OTUs were obtained from the single and double PCR experiments, respectively. To confirm these sequences were from organisms likely to occur in this environment, we used the blastn algorithm [34] to compare our sequences to the NCBI nucleotide database and report the results for the 10 most abundant OTUs of each treatment (S2 Table).

**Fig 2:**
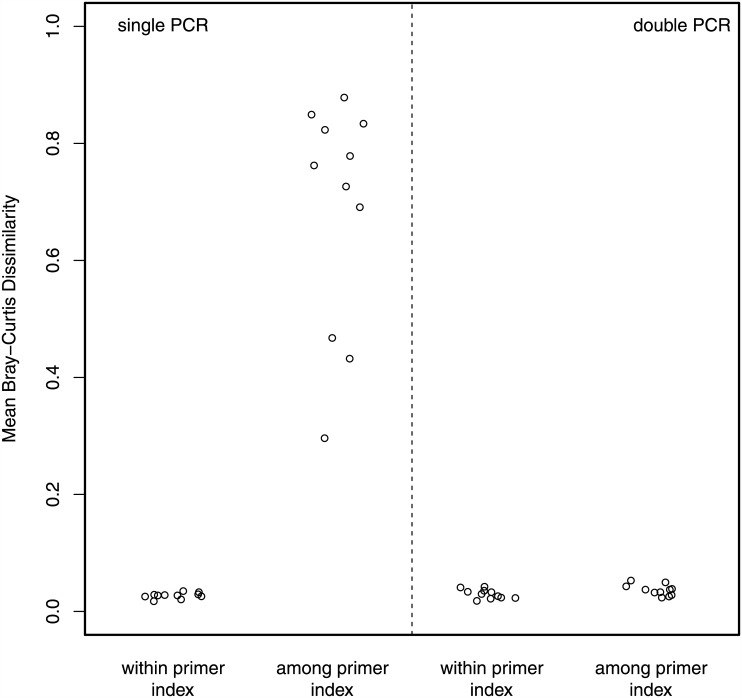
Dissimilarity among results from indexed primer sets used on the same environmental sample. Mean pairwise Bray-Curtis dissimilarity among sequencing replicates within and among indexed primer sets used to amplify each environmental sample (*N* = 11) using both a single (left) and double (right) PCR protocol. Bray-Curtis dissimilarity value of 0 indicates two samples are exactly identical while a value of 1 indicates they are exactly different. For the single PCR treatment, the mean Bray-Curtis dissimilarity values for within-primer index comparisons (*M* = 0.027, *SD* = 0.0050) were significantly lower than those of among-primer index comparisons (*M* = 0.685, *SD* = 0.196; Welch’s *t*(10.01) = 11.13, *p* < 0.00001).

For the single PCR treatment, there was little variation among OTU sequence counts generated with the same indexed primers (Bray-Curtis Dissimilarity; *M* = 0.027, *SD* = 0.0050; Fig 2). By contrast—and strikingly contrary to the assumption that primer indexes do not influence analytical outcomes—there were large differences between OTU counts generated using different indexed primer sets on the same environmental sample (Bray-Curtis Dissimilarity; *M* = 0.685, *SD* = 0.196) and these were significantly greater than comparisons within the same primer index (Welch’s *t*(10.01) = 11.13, *p* < 0.00001; Fig 2). Note that the low dissimilarity within primer index (and thus among library indexes) is indicative that ligated library indexes do not affect community-level measures.

**Fig 3:**
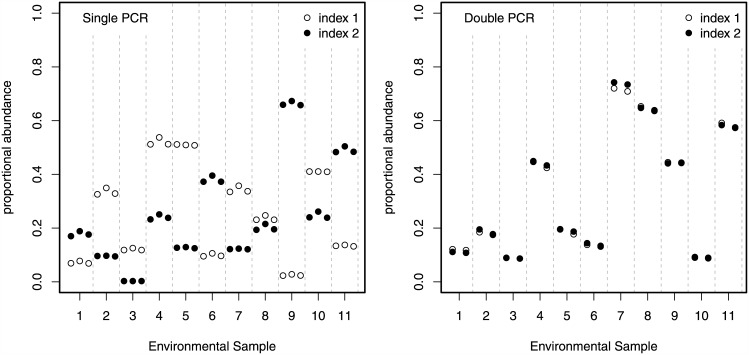
Effect of indexed primers on the proportional abundance of a representative OTU. Proportional sequence counts for a representative OTU (annotated to the gastropod genus *Elysia*) from each of 11 independent environmental samples (distinguished on the horizontal axis and delineated by vertical grey dashed bars). Each sample was amplified using two indexed primer sets (represented by closed or open points), each of which were sequenced in multiple replicates (single PCR *N* = 3; double PCR *N* = 2). Within a sample, variance among instances of the same symbol (among filled points or among closed points) represents variance across the three different sequencing libraries using the same indexed primer set. Variance between symbols (between open and closed points) indicates variance in relative abundance due to differences between primer indexes.

Primer indexes had large template sequence-specific effects in the single-PCR treatment. Ten OTUs comprised 80% of the total sequence data across all environmental samples. For these OTUs, the maximum difference in mean proportional abundance across primer index within a single environmental sample was 77.1% (OTU 2). For example, the mean abundance of the most abundant OTU (annotated as *Elysia*, a gastropod genus common in the sampled eelgrass habitat) varied between 2.3% and 67.3% within the same water sample, due almost entirely to differences in the 6bp multiplexing primer index (Fig 3).

There was no discernible effect of primer index bias using the double PCR approach. Dissimilarity among replicates within an environmental sample was low both within (*M* = 0.030, *SD* = 0.0081) and among (*M* = 0.0364, *SD* = 0.0093) primer index replicates. There was no difference in the mean dissimilarity between the within- and among-primer index comparisons using double PCR (Welch’s *t*-test, *p* = 0.092).

## Discussion

We found strong evidence that PCR primers that include an index sequence can bias the resulting sequence counts in mixed-template genomic samples, presumably via differential amplification efficiency among templates. A double-PCR approach eliminated the effects of bias caused by primer indexes. The first PCR using non-indexed primers generates amplicons that terminate in primer sequence at either end, thus precluding interaction between primer index and variable template in the second PCR. That is, amplicons produced using non-indexed primers lack a primer binding site at which the index sequence causes differential amplification among templates. We believe this effect is independent of amplicon size.

As amplification and parallel sequencing of mixed-template samples becomes an increasingly commonplace method for surveying biodiversity, understanding and eliminating potential sources of bias at the molecular level is imperative. Because high-throughput sequencing platforms provide vastly more per-sample sequencing depth than is necessary for many applications, multiplex sequence indexes are attractive for making large-scale molecular ecology more cost-effective. We have shown that when used in a single reaction, indexed PCR primers bias the resulting sequence count data on which many sequencing studies focus. Although the authors were very likely unaware of these potential biases, many published data sets may be subject to them, including comparative surveys of human microbiomes, ecological communities, and gene expression. The magnitude of this effect is surprisingly high, such that it is likely to dwarf any underlying signal in the data.

The indexed primer approach has become popular for its cost efficiency, and thus there is high potential for bias in existing data sets. However, the effect of the bias on the conclusions presented in manuscripts will vary depending on the inference drawn from the data set. Patterns inferred from the counts of a single sequence variant (e.g. OTU) are more likely to be erroneous, while studies focused on whole-community measures such as richness or diversity may not be as strongly affected. Additionally, where environmental and PCR sampling is even minimally replicated across primer indexes, the effect on final analyses is most likely to be that true patterns are obscured by noise introduced by different indexes on each sample. Likewise, every study using indexed primers in a single PCR is not necessarily flawed. Situations may exist where there is no genetic variation among the template sequences at the site where primer indexes bind, and sequencing through the primer binding site could confirm this on a case-by-case basis. We encourage readers to consider the methodological details of such studies when evaluating their conclusions.

We recommend that amplicon-based sequencing studies take one of two approaches to multiplex samples and improve sequencing efficiency. First, index sequences ligated onto amplicons post-PCR avoid the potential for amplification bias. Commercial library preparation kits include this as an option, which is more cost-effective than sequencing a single sample per run, but still more expensive than primer-based indexes. Second, a two-step PCR avoids index bias by first amplifying a sample of mixed template with non-indexed primers, and then using the resulting amplicons as template for a second PCR with indexed primers [23]. This avoids interaction between genomic template and the primer index, which is presumably responsible for the bias we observed.

Finally, our results highlight the importance of replication to test for variation at each of the levels of experimental design. Each step in the experimental process—from field sampling to sequencing—can introduce bias, which compounds along the chain of analysis. It is therefore critical to include sufficient replication in order to be able to apportion variance among these procedural steps. We therefore recommend that researchers plan for preliminary sequencing runs to assess this variance before attempting to describe biological phenomena using these techniques. However, we emphasize that we are not proposing that indexed primers are the only potential source of bias, or that bias can be averted by using double PCR or additional sequencing runs. Indeed, bias can be introduced at many stages in the workflow for high throughput sequencing studies (e.g. for environmental DNA metabarcoding see[35]), and we recommend investigators seek out and implement the strategies proposed to address these concerns. Recent advances include incorporation of site-occupancy detection models [36], accounting for primer bias [37], and environmental replication [38] among others.

## Supporting Information

S1 Table Primer table. Primers and primer index sequences (5′ to 3′) used in this study.
(CSV)

S2 Table Summary of results of taxonomic annotation. Sequences were queried against the full NCBI nucleotide database (nt) obtained on 28 September 2015, using the BLASTN algorithm with a word size of 7 with no restriction on the lower bound for percent identity. A maximum of 1000 hits were retained per query sequence; if match quality dropped after 100 sequences, matches were no longer retained. We used a nested approach whereby sequences were queried at sequentially higher e-value thresholds (i.e. inferior match) until a match was assigned. The e-value thresholds were determined based on a preliminary assessment of the best possible e-value given sequence length and database size (4.43e-52, 3.08e-48, 2.14e-44, 1.49e-40, 1.03e-36, 7.17e-33, 4.98e-29, 3.46e-25, 2.40e-21, 1.67e-17, 1.16e-13, 10). Thus, we report here the lowest taxonomic classification at which there was agreement among equally good matches.
(CSV)
